# A global map of planting years of plantations

**DOI:** 10.1038/s41597-022-01260-2

**Published:** 2022-04-01

**Authors:** Zhenrong Du, Le Yu, Jianyu Yang, Yidi Xu, Bin Chen, Shushi Peng, Tingting Zhang, Haohuan Fu, Nancy Harris, Peng Gong

**Affiliations:** 1 College of Land Science and Technology, China Agricultural University, Beijing, 100083 China; 2 Key Laboratory of Agricultural Land Quality, Ministry of Natural Resources of the People’s Republic of China, Beijing, 100083 China; 3 Ministry of Education Key Laboratory for Earth System Modeling, Department of Earth System Science, Tsinghua University, Beijing, 100084 China; 4 Ministry of Education Ecological Field Station for East Asia Migratory Birds, Tsinghua University, Beijing, 100084 China; 5 Division of Landscape Architecture, Faculty of Architecture, The University of Hong Kong, Hong Kong SAR, China; 6 Sino-French Institute for Earth System Science, College of Urban and Environmental Sciences, Peking University, Beijing, 100871 China; 7 World Resources Institute, Washington DC, USA; 8 Department of Geography and Department of Earth Sciences, The University of Hong Kong, Hong Kong SAR, China; 9 Institute for Climate and Carbon Neutrality, the University of Hong Kong, Hong Kong, China

**Keywords:** Forestry, Forestry

## Abstract

Plantation is an important land use type that differs from natural forests and affects the economy and the environment. Tree age is one of the key factors used to quantify the impact of plantations. However, there is a lack of datasets explicitly documenting the planting years of global plantations. Here we used time-series Landsat archive from 1982 to 2020 and the LandTrendr algorithm to generate global maps of planting years based on the global plantation extent products in Google Earth Engine (GEE) platform. The datasets developed in this study are in a GeoTIFF format with 30-meter spatial resolution by recording gridded specie types and planting years of global plantations. The derived dataset could be used for yield prediction of tree crops and social and ecological cost-benefit analysis of plantations.

## Abstract (summary)

**Table:** 

Measurement(s)	planting years of plantations
Technology Type(s)	remote sensing and LandTrendr algorithm
Sample Characteristic - Organism	forest

## Background & Summary

Forests are the dominant terrestrial ecosystem on Earth, covering approximately one-third of the global land area, and they play an important role in the water and heat balance, global carbon cycle and climate change^1–3^. Since the turn of the century, forest loss driven by human activities and natural disturbance, such as agricultural expansion, fire and pest infestation, has been a serious global environmental issue^4^. On the brink of the decrease in biodiversity, carbon storage and economic incomes caused by forest loss^5^, many countries have implemented tree planting programmes, which have resulted in the expansion of plantations around the world^6–8^.

Plantations, as distinct from natural forests, generally consist of tree crops and planted forests, where tree crops are planted to provide fruits, nuts or other tree products (such as coconut, coffee and oil palm), and planted forests are grown for the production of wood and wood fiber or for the protection of ecosystems. Plantations can not only bring economic benefits by supplying wood, fiber, fruit, nuts and other products, but also provide important ecosystem services such as carbon sequestration and biodiversity promotion^9^. However, some plantations (e.g., oil palm) have negative impacts on society and the environment^10^, biodiversity declines^11^, loss of natural forests^12^ and peatlands^13^, and carbon emissions^14^. The quantification of positive and negative outcomes from plantations, such as yield and aboveground biomass estimate, usually depend on the planting year or tree age because this directly determines the biophysical attributes, including canopy size, tree height, root structure, and soil properties^15–17^. Although regional and national tree age datasets/annual datasets for certain plantations have been developed recently^18–20^, the planting years of plantations remain unknown at the global scale. To forecast the supply and demand of tree crops and quantify the benefits or costs of planted forests, it is essential to generate and share global planting year maps for plantations.

Compared with traditional methods for estimating tree age, which are mainly based on the biophysical properties of sample trees (e.g., tree rings, height, or crown size)^21,22^, remote sensing has the advantages of high efficiency and low costs. The biophysical characteristics of trees, such as leaf area index (LAI) and tree height, can be estimated through remote sensing observations. By combining these with the tree species data, statistical relationships between these characteristics and tree age can be developed^23,24^. However, these empirical models are difficult to apply globally because they are usually generated locally for certain tree species and are not applicable to crop trees^25,26^. Recently, public access to Landsat imagery and the development of cloud-computing platforms have made it possible to use long-term remote sensing images to detect and monitor forest disturbances^27–29^. On the basis of the time series of Landsat data, temporal trajectory analysis algorithms, such as Breaks for Additive Season and Trend (BFAST)^30^ and Landsat-based Detection of Trends in Disturbance and Recovery (LandTrendr)^31^, have been proposed to make full use of the rich and continuous temporal information from Landsat imagery. Several recent studies have applied LandTrendr to monitor forest loss and gain^32–34^, proving that the temporal fitting and segmentation strategies in this algorithm are effective for the mapping of planting years.

**Fig. 1:**
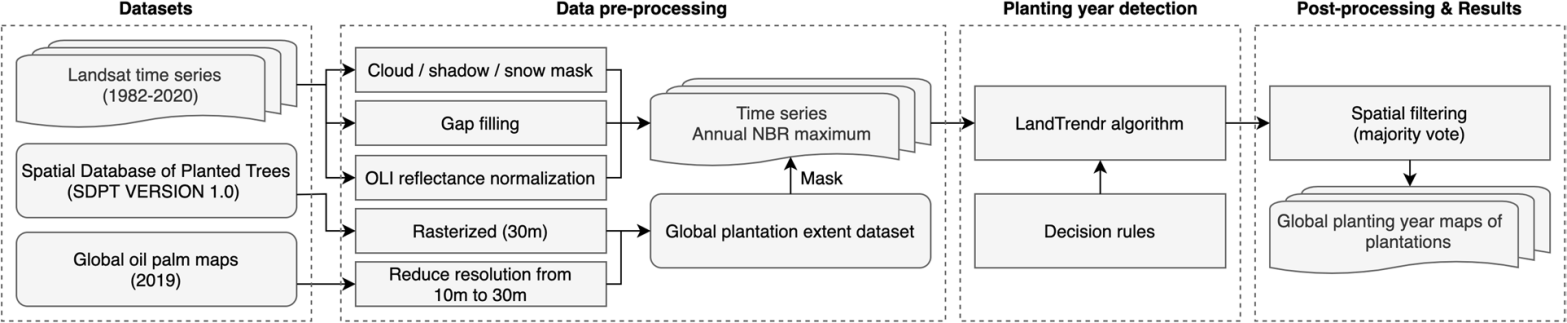
Workflow of generating global planting year maps of plantations.

In this study, we used the latest global plantation extent dataset to estimate and map their planting years at a 30 m resolution using Landsat imagery. The global plantation extent dataset was composed of the Spatial Database of Planted Trees (SDPT)^35^ and Descals’ global oil palm maps^36^, including planted forests and tree crops. We developed a method using the Google Earth Engine (GEE) to detect the year of the planting event with the LandTrendr algorithm and the time-series Landsat images spanning 1982–2020 (Fig. 1). We further included regional-scale planting year products and the first all-season sample set (FAST)^37^ for a comparison to evaluate the accuracy of the derived planting year map in this study.

## Methods

### Data preprocessing

#### Global plantation extent dataset

The SDPT provides global spatial information on planted forests and tree crops by compiling and synthesizing national or regional data. The product is an active database that is being gradually improved and enlarged. However, only version 1.0 of the product can be publicly downloaded. This version of the SDPT contains spatial data on plantations in countries shown in Table S1. The SDPT is a Geographic Information System (GIS) vector file with an attribute table that includes the original data source, species and polygon area. To improve the efficiency of planting year detection, the SDPT product was transformed into a raster with a resolution of 30 m. The information on the tree species in the product was retained by setting the value of the raster according to Table S2. The planting extent of oil palm in the SDPT was replaced by the latest global oil palm map provided by Descals *et al*.^36^ because it gives a more comprehensive insight into both smallholder and industrial oil palm plantations. The oil palm map is hosted on GEE as an *Image Collection* with a resolution of 10 m. To match the Landsat resolution, the resolution of the oil palm map was reduced to 30 m. The preprocessed SDPT raster and Descals’ oil palm map were used as a mask in the following process to identify the planting years.

#### Landsat imagery

All available Landsat Tier 1 surface reflectance imagery in GEE acquired from June to August between 1982 and 2020 was used in this study, including images collected by the Thematic Mapper (TM), Enhanced Thematic Mapper Plus (ETM+) and Operational Land Imager (OLI) sensors. We used images corresponding to the same season for each year to reduce the possibility of falsely detecting land-cover change because of phenology, flooding, or changes in solar geometry^31^. All the Landsat images were atmospherically corrected using Landsat ecosystem disturbance adaptive processing system (LEDAPS)^38^ and Landsat 8 Surface Reflectance Code (LaSRC)^39^ by the United States Geological Survey (USGS). Clouds, shadows, and snow were masked using the C Function of Mask (CFMask)^40^ algorithm. Considering the good agreement between the TM and ETM+^41–43^, we only normalized the OLI reflectance using the coefficients introduced in Roy’s paper^44^ to reduce mapping errors caused by the difference between the OLI and ETM+ sensors. Then, the annual maximum value of the Normalized Burn Ratio (NBR)^45^ was calculated to derive the time series for detecting the planting year. NBR is an index that uses a combination of near-infrared (*NIR*) and short-wave infrared (*SWIR*2) bands (Eq. (1)), and generally healthy and dense vegetation corresponds to a high NBR value. We chose NBR as the spectral index because it has a high sensitivity to forest disturbance and recovery^32,46^.

1 $$NBR=\frac{NIR-SWIR2}{NIR+SWIR2}$$

Owing to the uneven distribution of data in various regions of the world, Landsat data in different regions accumulated in different years, and some regions had missing data in some years. To generate a complete and continuous NBR time series from 1982 to 2020, we defined a 3-year sliding window that moved backwards from 2020. A null value in 2020 was supplemented by the value of the year closest to it. For other years where the NBR was null, we calculated the mean value of the 2 adjacent years to fill the gap. For years where the previous year was also null, we used the value of the following year as the supplementary value.

### Planting year detection using LandTrendr

To estimate the planting years, we used the LandTrendr algorithm, which was implemented on GEE^47^. LandTrendr obtains temporal segmentation results by analyzing the temporal-spectral trajectory of each pixel. The algorithm includes the following steps: 1) removing the ephemeral spikes; 2) identifying potential vertices by simple regression lines; 3) removing excess vertices based on low angle change; 4) choosing a single path through the vertices based on flexible fitting rules; 5) developing simplified models of the trajectory by removing the weakest vertices; 6) picking the model with the best fit (using p-value for the F-statistic). The parameter settings for LandTrendr in this paper followed the values used in De Jong’s study^34^, shown in Table 1. Taking the time series of NBR from 1982 to 2020 as the input, the output of LandTrendr was composed of four bands, including the observation year, the original observation value, the fitted observation value, and a Boolean value indicating whether an observation was identified as a vertex.

**Table 1:** Parameter settings for LandTrendr in this study.

LandTrendr parameters	Values
Max Segments	10
Spike Threshold	0.9
Vertex Count Overshoot	3
Prevent One Year Recovery	False
Recovery Threshold	1.0
pval Threshold	0.05
Best Model Proportion	0.75
Min Observations Needed	6

The definition for parameters can be found in the user manual (https://emapr.github.io/LT-GEE/index.html).

For each pixel, we aimed to identify the latest planting event in this study because more than one segment was detected for most pixels using LandTrendr. To remove false detection, we designed a set of decision rules: 1) the duration of the segment should be greater than 1 year; and 2) the delta of fitted observation value in the segment should be greater than 0.2. Then, the observation year of the start vertex was detected as the planting year. For pixels that had no segment meeting the decision rules, we detected the segment with the largest increase in NBR and took the start vertex as the planting year. For pixels that had no increase segment, the planting year was set as “<1982”.

LandTrendr is a change detection algorithm based on a pixel-by-pixel spectral time series. To reduce the effect of “salt and pepper”, a spatial filtering with a 3 × 3 window was applied to the mapping results based on a “majority vote” rule^48^. This resulted in global planting year maps for 1982–2019 from Landsat imagery between 1982 and 2020.

## Data Records

The dataset developed in this study can be downloaded on the figshare (10.6084/m9.figshare.19070084.v1)^49^. The dataset has a spatial resolution of 30 m. It is in a GeoTIFF format and has three bands named “plantyear”,“startyear” and “species”, which describe the planting years, the year in which Landsat TM, ETM+ and OLI data began to accumulate, and the species of trees. The “startyear” band should be used as the Quality Assessment (QA) band for this dataset because the supplementary value from 1982 to the start year may contain erroneous LandTrendr estimates of planting years.

The values of the three bands range from 1981 to 2019, 0 to 2020 and 1 to 190, respectively. For the “plantyear” band, a value of 1981 means the planting year was before 1982, and values from 1982 to 2019 correspond to the planting years. A value of 0 in the “startyear” band indicates that no Landsat data from June to September was obtained for the pixel during the period 1982–2020. Values from 1 to 1981 are not present and values 1982–2020 show the year when Landsat data started to accumulate. Values 1–190 in the “species” band represent tree species provided by the SDPT (Table S2).

**Fig. 2:**
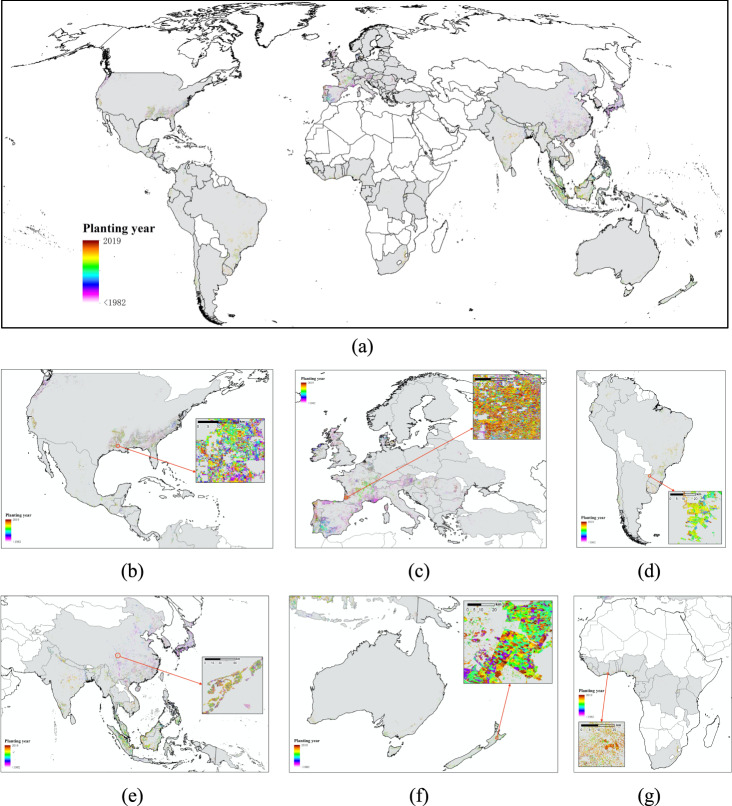
Planting year maps of (**a**) global plantations, and plantations in (**b**) North America, (**c**) Europe, (**d**) South America, (**e**) Asia, (**f**) Oceania and (**g**) Africa. (For more detailed visualization of results, please see: https://duzhenrong.users.earthengine.app/view/globalplantationyear).

Approximately 61.73% of plantation pixels have accumulated Landsat data since 1984, and we provide the annual extent of new plantations between 1984 and 2019 in Table 2. Figure 2 shows the planting years of global plantations, and Figure S1 provides information on the QA band of this dataset. To better show the results, the countries with plantation data are marked in grey.

**Table 2:** Extent of annual new plantations (1984–2019).

Year	Planted forest (km^2^)	Tree crop (km^2^)	Year	Planted forest (km^2^)	Tree crop (km^2^)
1984	38081.25	1658.16	2002	26899.13	14919.97
1985	38849.56	3148.84	2003	48680.67	6845.50
1986	46103.87	1913.93	2004	26928.43	11407.72
1987	39424.48	1753.86	2005	38878.37	12256.13
1988	47016.20	7270.16	2006	35041.45	13032.23
1989	46314.86	8292.65	2007	41681.92	13066.09
1990	33677.97	8078.22	2008	34745.10	10844.11
1991	34516.17	7699.37	2009	34129.27	14163.50
1992	47023.86	13293.75	2010	36073.25	13457.91
1993	38328.85	13775.80	2011	41392.27	12747.78
1994	34690.72	13254.14	2012	60099.08	16669.23
1995	36014.52	9022.56	2013	36089.22	11627.89
1996	32672.71	11336.06	2014	39440.88	12739.29
1997	37637.05	17848.83	2015	37609.67	11416.74
1998	39535.73	14817.48	2016	36046.63	9378.73
1999	39918.69	11810.74	2017	32888.45	6646.81
2000	31976.98	11119.38	2018	33712.34	7315.68
2001	24877.01	9288.99	2019	21001.37	4702.84

The external data used in this paper included the global plantation extent dataset and validation dataset. The global plantation extent dataset was composed of the SDPT^35^ and Descals’ oil palm map^36^, which can be downloaded at https://www.wri.org/research/spatial-database-planted-trees-sdpt-version-10 and 10.5281/zenodo.4473715, respectively. The validation dataset was composed of Danylo’s oil palm planting year product^20^, Chen’s orchard planting year product in California^19^ and FAST^37^ from https://dare.iiasa.ac.at/85/, 10.1016/j.isprsjprs.2019.03.012 and 10.1016/j.scib.2017.03.011, respectively.

## Technical Validation

### Validation with the oil palm planting year product

**Fig. 3:**
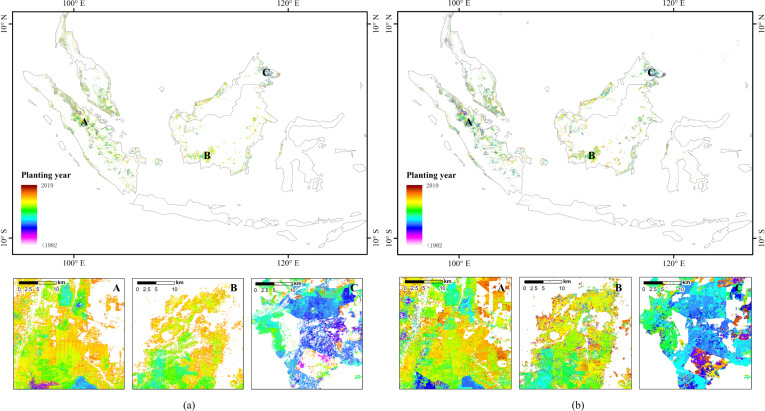
Comparison with oil palm planting year product in Indonesia, Malaysia, and Thailand. (**a**) Maps of Danylo’s product^20^. (**b**) Maps of our results.

We validated our results using Danylo’s oil palm planting year product in Indonesia, Malaysia and Thailand^20^. The product has a spatial resolution of 30 m with a single band that identifies the planting year of oil palm between 1987 and 2017. The definition of planting year for the product differed from ours, which said that the detected planting year was 2–3 years behind the plantation. Therefore, we subtracted 3 years from the value in the Danylo product to match our results. The comparisons are shown in Fig. 3. Although the distribution range of oil palm is different, the two datasets still show a satisfactory consistency in the planting years.

**Fig. 4:**
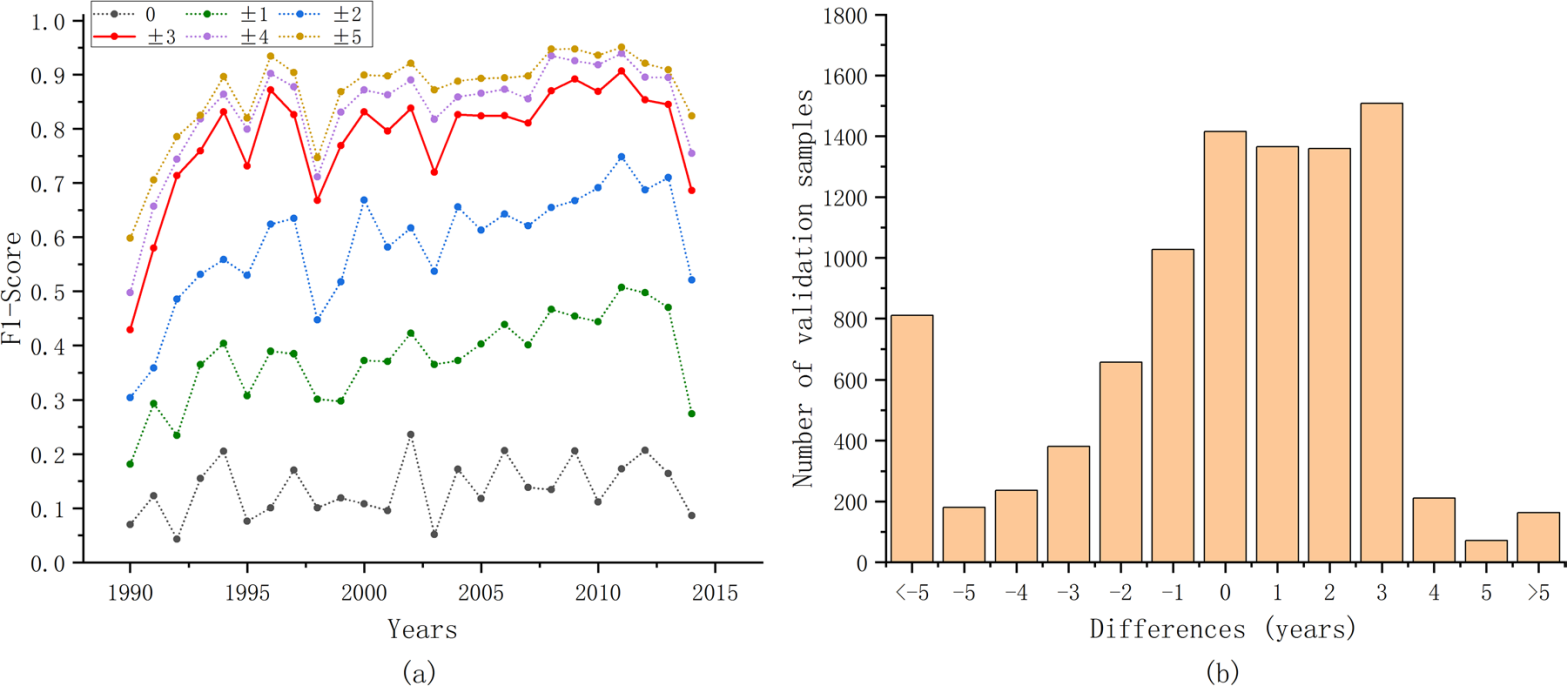
Accuracy evaluation results with oil palm planting year product in Indonesia, Malaysia and Thailand. (**a**) Annual F1 score with different tolerances. (**b**) Differences between our results and Danylo’s product^20^. The horizontal axis represents the value of the planting years in our results minus that in Danylo’s product^20^.

To obtain quantitative accuracy evaluation results, the annual F1 score was calculated. First, because the extent of oil palm in Danylo’s product differs from the extent used in our study, we extracted the areas that overlapped between the two maps. We vectorized the overlapping area, and selected polygons larger than 10 hectares to randomly generate validation samples. Then, using 10,000 validation samples, we calculated the F1 score for each year (Fig. 4(a)). Considering the sparsity of the pre-1990 Landsat data and the possible uncertainties arising from it, accuracy validation results after 1990 was provided here. The F1 score for each year was relatively high (78.25%) when the deviation was allowed within ±3 years and 86.83% with ±5 years. Our method tended to identify the planting year later than the Danylo product (Fig. 4(b)). This was probably induced by the differences in the definition of planting years in the two studies.

### Validation with orchard planting year product in California

**Fig. 5:**
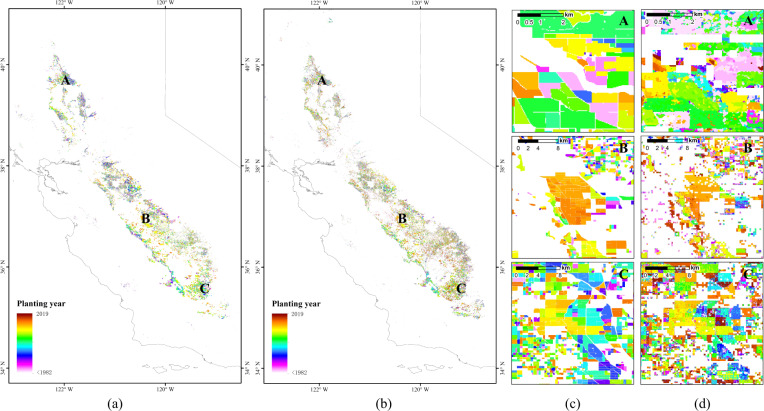
Comparison with orchard planting year product in California. (**a**) and (**c**) are maps of Chen’s product^19^. (**b**) and (**d**) are maps of our results.

An orchard-level planting year product of all fruit and nut trees in California provided in Chen’s study^19^ was also used to validate our mapping result. The product was developed based on the geographic boundaries for all nut and fruit tree orchard blocks from the 2014 state-wide Crop Mapping dataset (https://gis.water.ca.gov/app/CADWRLandUseViewer/). Planting years were identified using Landsat imagery from 1984 to 2014. The comparison between the Chen product and our results are shown in Fig. 5. Because the planting years in the product were developed based on the mean and standard deviations of NDVI within each block, Chen’s product has better space contiguity. However, judging from the overall distribution and the zoomed-in details, the results of this study have a high consistency with the orchard planting year product in California.

**Fig. 6:**
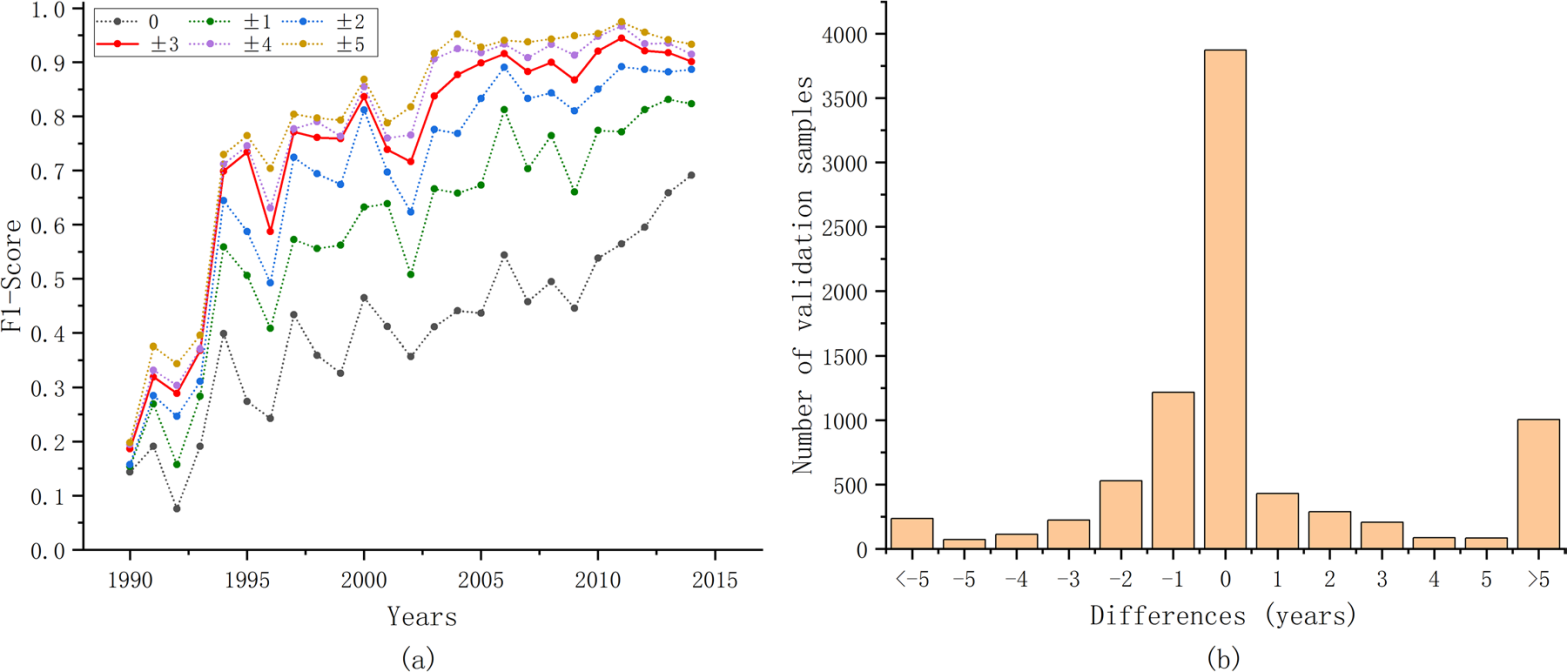
Accuracy evaluation results with orchard planting year product in California. (**a**) Annual F1 score with different tolerances. (**b**) Differences between our results and Chen’s product^19^. The horizontal axis represents the value of the planting years in our results minus that in Chen’s product^19^.

To further quantify the consistency, validation samples were generated using the same method mentioned in the previous section. The annual F1 score and differences in planting years between our results and the Chen product were calculated (Fig. 6). Generally, the annual F1 score increased with age, and our results achieved satisfactory accuracy when the tolerance was ±3 years.

### Validation with the FAST

**Fig. 7:**
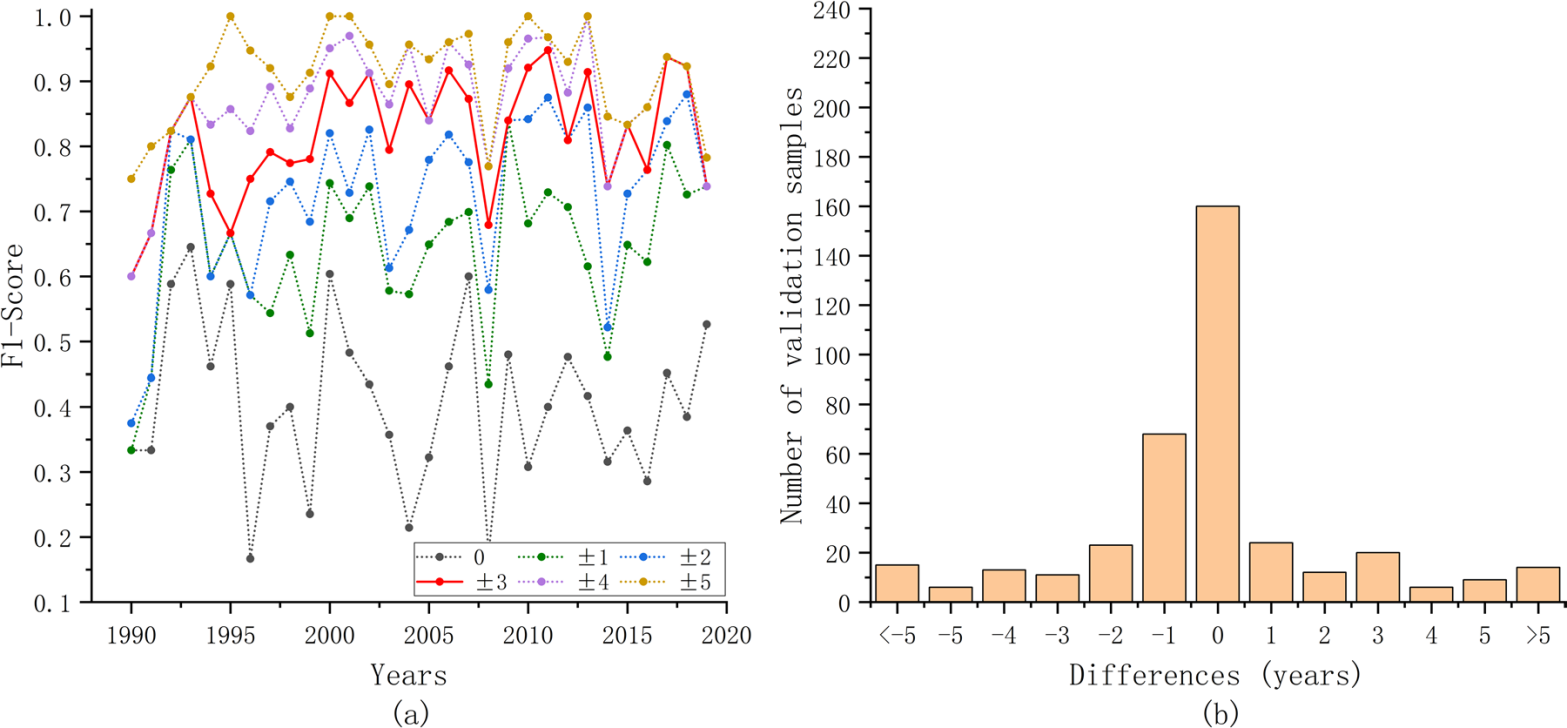
Accuracy evaluation results with the first all-season sample set (FAST)^37^. (**a**) Annual F1 score with different tolerances. (**b**) Differences between our results and visually interpreted labels. The horizontal axis represents the value of the planting years in our results minus visually interpreted labels.

To give the relatively comprehensive verification of our results, the FAST^37^ in the Finer Resolution Observation and Monitoring of Global Land Cover (FROM-GLC)^50^ project was used for the validation. Both training and validation samples labeled forest and located in the plantation extent in FAST were selected as the validation samples. Planting years of the 757 validation samples were labeled using Huang’s sample migration method^51^. The spectral similarity and distance between the reference year and target year was measured. The planting years of the validation samples were marked as the year with the greatest spectral difference, and checked artificially based on the visually interpretation of Landsat images. The annual F1 score and differences in planting years between our results and visually interpreted labels (after 1990) were shown in the Fig. 7. Our results still show a high accuracy with a tolerance of ±3 years, and remarkable accuracy could be obtained when the tolerance was ±5 years.

## Usage Notes

The planting year maps developed in this study are the first high-resolution dataset for global plantations at a 30 m spatial resolution. When overlayed with other global or local climate and socio-economic data, the dataset can be used to evaluate the social and ecological benefits and costs from plantations, such as the carbon sequestration from planted forests. Because this dataset contains information on the tree species, it can also help with the prediction of yield from tree crops and the demand for water. Our code to map planting years can be reused on other similar plantation datasets, such as a new version of the SDPT, which may be released in the future. However, parameter settings for LandTrendr in this study were determined based on the previous research and the testing results for NBR time series of the plantations. When applying this method to other vegetation restoration detections, such as the natural restoration of forests, these parameters may need to be adjusted. In addition, users should be aware of the strips in the dataset which are caused by the banding problem in Landsat ETM + images.

Our dataset is in a GeoTIFF format, and thus can be easily loaded and processed by any GIS software, such as QGIS and ArcGIS. The dataset can be visualized online using the GEE experimental app: https://duzhenrong.users.earthengine.app/view/globalplantationyear. It should be noted that to optimize the visualization effect, we merged plantations with mixed species into one category in this app; for example, “Acacia/Wattle, Eucalyptus” and “Acacia/Wattle, Kirkii” were merged as “Acacia/Wattle”.

## Supplementary information

Supplementary Information

## Data Availability

The GEE code and validation samples in this study are available in GitHub at https://github.com/IrisDudu/globalplantations.
