# Supplementary material for: A global map of planting years of plantations
Source: Sci Data. 2022 Apr 1;9:141. doi: 10.1038/s41597-022-01260-2 (PMC8975943; doi:10.1038/s41597-022-01260-2)
Supplement: Supplementary file 1 — Supplementary Information [file 41597_2022_1260_MOESM1_ESM.pdf]

Supplementary
A global map of planting years of plantations

Zhenrong Du[1,2,3], Le Yu[3,4,*], Jianyu Yang[1,2,*], Yidi Xu[3], Bin Chen[5], Shushi Peng[6], Tingting Zhang[1,2], Haohuan Fu[3], Nancy Harris[7], and Peng Gong[4,8,9]

[1]College of Land Science and Technology, China Agricultural University, Beijing 100083, China.
[2]Key Laboratory of Agricultural Land Quality, Ministry of Natural Resources of the People's Republic of China, Beijing 100083, China.
[3]Ministry of Education Key Laboratory for Earth System Modeling, Department of Earth System Science, Tsinghua University, Beijing 100084, China.
[4]Ministry of Education Ecological Field Station for East Asia Migratory Birds, Tsinghua University, Beijing 100084, China.
[5]Division of Landscape Architecture, Faculty of Architecture, The University of Hong Kong, Hong Kong SAR, China.
[6]Sino-French Institute for Earth System Science, College of Urban and Environmental Sciences, Peking University, Beijing 100871, China.
[7]World Resources Institute, Washington DC, USA.
[8]Department of Geography and Department of Earth Sciences, The University of Hong Kong, Hong Kong SAR, China.
[9]Institute for Climate and Carbon Neutrality, the University of Hong Kong, Hong Kong, China.

[*]corresponding author(s): Le Yu (leyu@tsinghua.edu.cn); Jianyu Yang (ycjyyang@cau.edu.cn)

Table of contents

No.	Title
Figure S1	The year where Landsat TM, ETM+ and OLI imagery started to be available.
Table S1	Countries and the data source year in Spatial Database of Planted Trees (SDPT) product.
Table S2	Correspondence between tree species and band value.

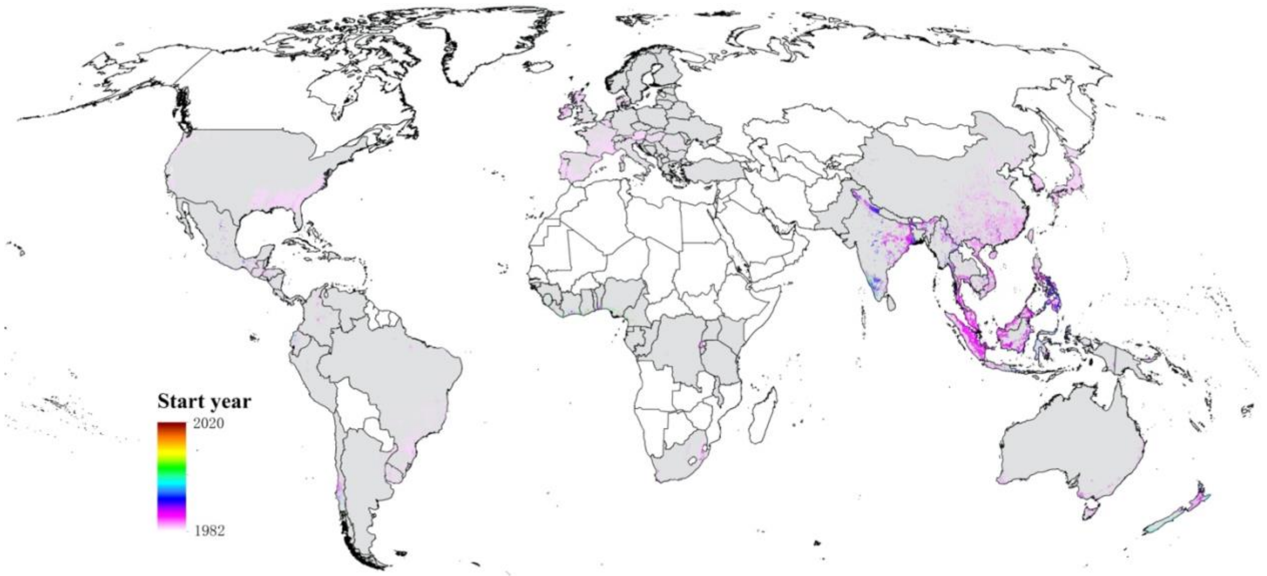

Figure S1. The year where Landsat TM, ETM+ and OLI imagery started to be available.

Table S1. Countries and the data source year in Spatial Database of Planted Trees (SDPT) product.

Country	Source year	Country	Source year
Argentina	2013	Malaysia	2013-2015
Australia	2013	Mexico	2011-2014
Brazil	2013/2014	Myanmar	2002 and 2014
Cambodia	2013/2014	Nepal	2015
Cameroon	Unknown	New Zealand	2012
Chile*	2016	Nicaragua	2014
China	2004-2008	Nigeria	2013-2015
Colombia	2013/2014	Pakistan	2015
Costa Rica	2012	Panama	2014
Congo, Dem. Rep. of	2013	Papua New Guinea	2012/2015
Ecuador	2000, 2014	Peru	2013/2014
Europe	2011, 2017	Philippines	2003, 2017
Gabon	2013-2015	Rwanda	2008
Ghana	2013-2015	Solomon Islands	2013-2015
Guatermala	2012	South Africa	2013/2014
Honduras	2013	South Korea	Unknown
India	2015	Sri Lanka	2013-2015
Indonesia	2013-2015	Thailand	2000
Ivory Coast	2013-2015	Uruguay	2015
Japan	1995-1999	United States	2008/2011, 2014/2016
Kenya	2010	Venezuela	2014
Liberia	2014	Vietnam	2016
Malawi	2012/2015		

[1]Source: Compiled by SDPT authors
[2]Note: *Data includes only 40% of total plantation area

Table S2. Correspondence between tree species and band value.

Band value	Tree species	Agricultural tree or planted forest
1	Acacia/Wattle	Planted forest
2	Acacia/Wattle mix	Planted forest
3	Acacia/Wattle, Eucalyptus	Planted forest
4	Acacia/Wattle, Kirkii	Planted forest
5	Acacia/Wattle, Teak	Planted forest
6	African Pear	Tree crop
7	Albizia Mix	Planted forest
8	Alder	Planted forest
9	Alder, Grevillea	Planted forest
10	Almond	Tree crop
11	Apple	Tree crop
12	Apricot	Tree crop
13	Areca Palm	Tree crop
14	Ash	Planted forest
15	Australian Blackwood	Planted forest
16	Bamboo	Planted forest
17	Bamboo, Eucalyptus, Black Wattle	Planted forest
18	Banana	Tree crop
19	Black Locust	Planted forest
20	Black Pine	Planted forest
21	Black Spruce	Planted forest
22	Black Wattle	Planted forest
23	Bocote	Planted forest
24	Cacao	Tree crop
25	Cashew	Tree crop
26	Casuarina	Planted forest
27	Cedar	Planted forest
28	Cherry	Tree crop
29	Chilean Cedar	Planted forest
30	Chinese Cork Oak	Tree crop
31	Christmas Tree	Tree crop
32	Citrus	Tree crop
33	Clove	Tree crop
34	Coconut Palm	Tree crop
35	Coconut Palm Mix	Tree crop
36	Coconut Palm, Areca Palm	Tree crop
37	Coconut Palm, Fruit	Tree crop
38	Coffee	Tree crop
39	Coffee Mix	Tree crop

Band value	Tree species	Agricultural tree or planted forest
40	Coffee, Acacia/Wattle	Tree crop
41	Coffee, Albizia	Tree crop
42	Coffee, Oil Palm	Tree crop
43	Cypress	Planted forest
44	Cypress Mix	Planted forest
45	Cypress Pine	Planted forest
46	Cypress Pine, Eucalyptus	Planted forest
47	Cypress Pine, Eucalyptus, Pine	Planted forest
48	Cypress Pine, Pine	Planted forest
49	Cypress, Eucalyptus	Planted forest
50	Cypress, Grevillea, Eucalyptus	Planted forest
51	Cypress, Pine	Planted forest
52	Douglas Fir	Planted forest
53	East Asian Ash	Planted forest
54	East Asian Eurya	Planted forest
55	East Asian White Birch	Planted forest
56	Eucalyptus	Planted forest
57	Eucalyptus, Australian Blackwood	Planted forest
58	Eucalyptus, Bamboo	Planted forest
59	Eucalyptus, Bamboo, Umbrella Tree	Planted forest
60	Eucalyptus, Black Wattle	Planted forest
61	Eucalyptus, Cypress Pine, Cypress	Planted forest
62	Eucalyptus, Cypress Pine	Planted forest
63	Eucalyptus, Grevillea	Planted forest
64	Eucalyptus, Grevillea, Pine	Planted forest
65	Eucalyptus, Pine	Planted forest
66	Eucalyptus, Pine, Alder	Planted forest
67	Eucalyptus, Pine, Australian Blackwood	Planted forest
68	Eucalyptus, Pine, Bamboo	Planted forest
69	Eucalyptus, Pine, Bamboo, Umbrella Tree	Planted forest
70	Eucalyptus, Pine, Cypress	Planted forest
71	Eucalyptus, Pine, Grevillea, Cypress	Planted forest
72	Eucalyptus, Umbrella Tree	Planted forest
73	Eucalyptus, Umbrella Tree, Pine	Planted forest
74	Fir	Planted forest
75	Fruit	Tree crop
76	Fruit Mix	Tree crop
77	Fruit, Areca Palm	Tree crop
78	Fruit, Cacao	Tree crop
79	Fruit, Oil Palm	Tree crop

Band value	Tree species	Agricultural tree or planted forest
80	Ginkgo	Tree crop
81	Gleditsia	Planted forest
82	Gliricidia	Tree crop
83	Grevillea	Planted forest
84	Grevillea, Cypress	Planted forest
85	Grevillea, Cypress Pine	Planted forest
86	Grevillea, Pine	Planted forest
87	Grevillea, Pine, Cypress Pine, Cypress	Planted forest
88	Grevillea, Senna	Planted forest
89	Jacaranda	Planted forest
90	Jacaranda, Eucalyptus	Planted forest
91	Jacaranda, Eucalyptus, Spectacular Cassia	Planted forest
92	Japanese Bay Tree	Planted forest
93	Japanese Black Pine	Planted forest
94	Japanese Flowering Cherry	Tree crop
95	Japanese Rasin	Planted forest
96	Japanese Red Cedar	Planted forest
97	Japanese Red Cedar, Hinoki Cypress, Sawara Cypress	Planted forest
98	Japanese Red Pine	Planted forest
99	Japanese Stone Oak	Tree crop
100	Jezo Spruce	Planted forest
101	Korean Chestnut	Tree crop
102	Korean Dendropanax	Tree crop
103	Korean Pine	Planted forest
104	Korean Red Pine	Planted forest
105	Larch	Planted forest
106	Loblolly Pine	Planted forest
107	Loblolly Pine Mix	Planted forest
108	Loose-Flower Honbeam	Planted forest
109	Mahogany	Planted forest
110	Mango	Tree crop
111	Mangrove	Planted forest
112	Mesquite	Planted forest
113	Monkey Puzzel	Planted forest
114	Mono Maple	Planted forest
115	Monterey Pine	Planted forest
116	Monterey Pine, Shining Gum	Planted forest
117	Monterey Pine, Tasmanian Bluegum	Planted forest
118	Mulberry	Tree crop
119	Nectarine	Tree crop

Band value	Tree species	Agricultural tree or planted forest
120	Oak	Planted forest
121	Oil Palm	Tree crop
122	Olive	Tree crop
123	Orange	Tree crop
124	Padauk	Planted forest
125	Palm	Tree crop
126	Palm, Coconut Palm	Tree crop
127	Palm, Fruit	Tree crop
128	Peach	Tree crop
129	Pear	Tree crop
130	Pecan	Tree crop
131	Peltophorum	Planted forest
132	Pine	Planted forest
133	Pine Mix	Planted forest
134	Pine, Acacia/Wattle	Planted forest
135	Pine, Eucalyptus, Acacia/Wattle, Cypress	Planted forest
136	Pistachio	Tree crop
137	Pitch Pine	Planted forest
138	Plum	Tree crop
139	Pomegranate	Tree crop
140	Poplar	Planted forest
141	Poplar, Willow	Planted forest
142	Princess	Planted forest
143	Red Cedar	Planted forest
144	Roble	Planted forest
145	Rosewood	Planted forest
146	Rubber	Tree crop
147	Rubber Mix	Tree crop
148	Rubber, Acacia/Wattle	Tree crop
149	Rubber, Areca Palm	Tree crop
150	Rubber, Coconut Palm	Tree crop
151	Rubber, Coffee	Tree crop
152	Rubber, Eucalyptus	Tree crop
153	Rubber, Fruit	Tree crop
154	Rubber, Mango	Tree crop
155	Rubber, Oil Palm	Tree crop
156	Rubber, Pine	Tree crop
157	Rubber, Teak	Tree crop
158	Sakhalin Fir	Planted forest
159	Sakhalin Spruce	Planted forest

Band value	Tree species	Agricultural tree or planted forest
160	Sal	Planted forest
161	Sawleaf Zelkova	Planted forest
162	Sawtooth Oak	Planted forest
163	Senna	Planted forest
164	Shining Gum	Planted forest
165	Shortleaf Pine	Planted forest
166	Shortleaf Pine Mix	Planted forest
167	Slash Pine	Planted forest
168	Slash Pine Mix	Planted forest
169	Southern Beech	Planted forest
170	Spanish Cedar	Planted forest
171	Spruce	Planted forest
172	Tasmanian Bluegum	Planted forest
173	Tasmanian Bluegum, Shining Gum	Planted forest
174	Taxodium	Planted forest
175	Tea	Tree crop
176	Teak	Planted forest
177	Teak Mix	Planted forest
178	Teak, Cadam	Planted forest
179	Tulip Poplar, Tulip Magnolia	Tree crop
180	Umbrella Tree	Planted forest
181	Unknown	Planted forest
182	Unknown	Tree crop
183	Walnut	Tree crop
184	Walnut, Spruce	Tree crop
185	Wedding Cake	Tree crop
186	White Cedar	Planted forest
187	Willow	Planted forest
188	Needleleaf	Planted forest
189	Mix of needleleaf and broadleaf	Planted forest
190	Broadleaf	Planted forest
